# The Effect of including a Mixed-Enzyme Product in Broiler Diets on Performance, Metabolizable Energy, Phosphorus and Calcium Retention

**DOI:** 10.3390/ani14020328

**Published:** 2024-01-21

**Authors:** Harriet Walker, Suvi Vartiainen, Juha Apajalahti, Jules Taylor-Pickard, Ivana Nikodinoska, Colm A. Moran

**Affiliations:** 1 Solutions Deployment Team, Alltech (UK) Ltd., Stamford PE9 1TZ, UK; harriet.walker@alltech.com (H.W.); jpickard@alltech.com (J.T.-P.); 2 Alimetrics Research Ltd., 02920 Espoo, Finland; suvi.vartiainen@alimetrics.com (S.V.); j.apajalahti@alimetrics.com (J.A.); 3 Alltech’s European Bioscience Centre, Dunboyne, A86 X006 Co. Meath, Ireland; ivana.nikodinoska@alltech.com; 4 Alltech SARL, Rue Charles Amand, 14500 Vire, France

**Keywords:** SSF, phytase, xylanase, digestibility, broiler, performance

## Abstract

**Simple Summary:**

Inorganic minerals are often provided in excess in poultry diets to meet the nutritional needs of the birds, with phosphorus and calcium being of particular importance. Phosphorus, which is not easily digestible by birds, is already present in many of the common plant-based ingredients in poultry diets. This plant-based phosphorus can be made more digestible for birds through the inclusion of enzymes such as phytase. Other enzymes such as xylanase can also be included to help breakdown the plant-based material and release essential minerals and nutrients. In this study, the effects of adding a natural enzyme complex with primary activity of phytase and secondary activity of xylanase, pectinase, protease, β-glucanase, cellulase and amylase to broiler chicken diets were tested. The enzyme-supplemented birds showed improvements in various digestibility parameters. More phosphorus was retained in the enzyme-supplemented diets when compared to the positive control. These findings indicate that these enzymes can be used to improve the efficient use of phytate-bound phosphorus without negatively affecting broiler productivity. The use of enzymes also reduced phosphorus excretion, therefore reducing the potential negative impacts on the environment.

**Abstract:**

The importance of enzymes in the poultry industry is ever increasing because they help to extract as many nutrients as possible from the raw material available and reduce environmental impacts. Therefore, an experiment was conducted to examine the effect of a natural enzyme complex (ASC) on diets low in AME, Ca and P. Male Ross 308 broilers (*n* = 900) were fed one of four diets: (1) positive control (PC) with no enzyme added (AME 12.55 MJ/kg, AVPhos 4.8 g/kg and AVCal 9.6 g/kg); (2) negative control (NC) with no enzyme added and reduced AME, Ca and P (AME 12.18 MJ/kg, AVPhos 3.3 g/kg, AVCal 8.1 g/kg); (3) negative control plus ASC at 200 g/t; and (4) negative control plus ASC at 400 g/t. Broiler performance, digesta viscosity, tibia mineralization and mineral content were analyzed at d 21. Between d 18 and 20, excreted DM, GE, total nitrogen, Ca, and P were analyzed. ASC at 200 g/t and 400 g/t improved the FCR (*p* = 0.0014) significantly when compared with that of the NC. There were no significant differences in BW or FI between the treatments. Birds fed ASC at 200 g/t and 400 g/t had significantly improved digesta viscosity (*p* < 0.0001) compared with that of the PC and NC birds and had significantly higher excreted DM digestibility (*p* < 0.01) than the NC and the PC birds with 400 g/t ASC. ASC inclusion significantly improved P retention (*p* < 0.0001) compared to that in the PC. Ca retention was significantly increased by 400 g/t ASC compared to that in the PC and NC (*p* < 0.001). AME was significantly higher (*p* < 0.0001) for all treatments compared to that in the NC. There were no significant differences between treatments for any of the bone measurements. This study showed that feeding with ASC can support the performance of broilers when fed a diet formulated to have reduced Ca, P and AME, with the greatest results being seen with a higher level of ASC inclusion.

## 1. Introduction

A range of exogenous enzymes are routinely added to broiler diets, and they play an essential role in poultry nutrition; the use of enzymes in poultry diets has been well established over the past three decades [1,2]. Their importance in the poultry industry is ever increasing with fluctuating feed raw material prices, the need to extract as many nutrients as possible from the raw material available and the need to reduce environmental impacts [3]. Both single- and multiple-activity products are often tailored to specific uses, such as targeting feed ingredients to increase digestibility and reduce the effect of their anti-nutritional factors, as well as to reduce waste and prevent pollution. There are many enzymes available to nutritionists, and the enzymes’ mechanisms of action and the beneficial effects of reducing anti-nutritional factors and increasing the energy, amino acids, mineral and other nutrient contents in animal feed have been well characterized [1,4]. Two of the main enzyme classes that are routinely used in poultry diets include non-starch polysaccharide enzymes and phytases. The estimated Feed Market Size for phytase alone was valued at USD 592.5 million in 2022 [5].

Non-starch polysaccharide (NSP) enzymes are used in poultry diets to break down the NSPs found in plant cell walls; this mainly includes pentoses, arabinoxylans and β-glucans in cereal grains [6]. NSP enzymes improve performance by reducing the digesta viscosity caused by NSPs and by making nutrients encapsulated by the plant cell wall available to the bird [7,8]. Increased digesta viscosity is caused by the NSPs in wheat and other raw materials absorbing water. This results in the reduced digestion and absorption of nutrients, as increased viscosity reduces digestive enzyme activity and the movement of nutrients [9,10]. More recently, the addition of the NSP enzyme xylanase has been linked to improved energy and nutrient availability and growth performance through the digestion of xylan into xylo-oligosaccharides (XOSs) [2,11,12]. XOS is a prebiotic that has been shown to have a beneficial effect on the gut by modulating microorganisms and immunity. The release of XOS can increase short-chain fatty acid (SCFA) production through promoting the growth of lactate- and butyrate-producing microorganisms in the gut [13,14,15]. Butyrate can be used by enterocytes as an energy source and help epithelial development, which is essential in the maintenance of normal intestinal barrier functions [16,17]. SCFAs are vital for birds to have an optimal gut function, microbiome, immunity and performance [12,18,19,20].

A large proportion of the phosphorus (P) in poultry feed raw materials from plant origins is not available for the bird to utilize, as it is bound to phytate, the principal storage form of P in plants. The low availability of plant P to poultry is due to monogastric species not being able to produce an endogenous enzyme that can release the P bound in phytate [21]. Therefore, to reduce the addition of inorganic P, the excretion of P in the litter and feed costs, phytase enzymes are added to broiler diets to liberate phytate-bound P. Phytases are not only added to poultry diets to improve the economics and reduce P inclusion and environmental impacts, they are also used to lower the chances of phytate becoming chelated with cations, such as calcium (Ca), zinc, iron and manganese, and to lessen the solubility and bioavailability of amino acids, leading to better performance with phytase inclusion [8,22].

This study investigated the effect of a natural enzyme complex, Allzyme Spectrum (ASC) (Alltech Inc., Nicholasville, KY, USA), which is produced via solid-state fermentation, using a naturally selected (non-GMO) strain of *Aspergillus niger* to produce an enzyme complex with primary activity of phytase and secondary activity of xylanase, pectinase, protease, β-glucanase, cellulase and amylase. Individually, these enzymes have been added to poultry diets to improve digestibility, reduce the effects of anti-nutritional factors and improve the performance of broilers. The following trial was conducted to investigate the effect of ASC, an enzyme complex supplementation in broiler diets from 1 to 21 d of age, to evaluate the effects of ASC on broiler performance and the digestibility of the experimental diets including metabolizable energy, phosphorus and calcium retention.

## 2. Materials and Methods

### 2.1. Birds and Husbandry

This feeding trial with broiler chickens was conducted in the licensed research facility of Alimetrics Research Ltd. in Southern Finland, in accordance with EU Directive 2010/63/EU [23] pertaining to the protection of animals used for experimental or other scientific purposes. A total of 900 day-of-hatch Ross 308 male broilers were randomized by weight and placed in 1.125 m^2^ floor pens in groups of 15 bedded on clean wood shavings. The maximum stocking density of 33 kg/m^2^ specified by welfare law was not exceeded over the rearing period. Birds were allowed ad libitum access to the treatment diets and water for the duration of the trial. The room was thermostatically controlled to produce an initial temperature of 32 °C on d 1, which was reduced during the study to 22 °C by d 21. The lighting regimen used was 24 h light on d 1 with increasing darkness for a week until 6 h of darkness was reached, which was maintained throughout the remainder of the study. All birds sampled were euthanized by cervical dislocation. Guidelines for the care and use of animals were followed and all experimental procedures involving animals were approved.

### 2.2. Dietary Treatment

Experimental diets were formulated to be nutritionally similar for each treatment (Table 1) except for AME, Ca and P which were reduced by altering soybean oil, limestone, and monocalcium phosphate to achieve the desired differences. Birds were allocated to 1 of 4 treatments, with each treatment replicated with 15 pens of 15 birds. Experimental diets: (1) positive control (PC) (no enzyme added, AME 12.55 MJ/kg, AVPhos 4.8 g/kg, AVCal 9.6 g/kg); (2) negative control (NC) with reduced AME, Ca and P (no enzyme added, AME 12.18 MJ/kg, AVPhos 3.3 g/kg, AVCal 8.1 g/kg); (3) NC plus ASC at 200 g/t; (4) NC plus ASC at 400 g/t. The treatment diets were a combination of wheat-, corn- and soybean-meal-based mash diets formulated to be comparable to the 2023 Ross 308 nutritional specifications. Calculated values for each experimental diet are shown in Table 2. The enzyme complex ASC (Allzyme^®^ Spectrum, Alltech Inc., Kentucky, KY, USA) is composed of Allzyme SSF from *Aspergillus niger* (NCIMB 30289) with primary activity of phytase (1000 SPU/g) and secondary activities of pectinase (4088 AJDU/g), protease (717 HUT/g), phytase (1000 SPU/g), β-glucanase (222 BGU/g), cellulase (41 CMCU/g) and amylase (30 FAU/g), and fortified with endo-1,4-b-xylanase (7500 EPU/g) produced by *Trichoderma citrinoviride* Bisset (IMI SD135). One solid-state fermentation phytase unit (SPU) is defined as the amount of enzyme required to liberate 1 μmol of inorganic phosphate per minute at pH 5.5 and 50 °C [24]. EPU is defined as the amount of enzyme which releases 0.0083 μmol of reducing sugars (xylose equivalent) per minute from oat spelt xylan at pH 4.7 and 50 °C [25]. Each experimental feed was mixed in one batch. Feed samples were collected for chemical analyses of dry matter (DM), titanium, Ca, P and gross energy (GE) (See Supplementary Table S1 Analytical composition of the diets). The diets were manufactured by Alimetrics Research Ltd. (Espoo, Finland) and included 4 kg/t titanium dioxide in feed as a marker for digestibility in all diets. The titanium and DM content of fecal samples were analyzed by Alimetrics Research Ltd. (Espoo, Finland).

An Alimetrics veterinarian inspected the health of the chicks at the beginning of the trial. During the trial, birds were observed twice a day and any with compromised health were excluded from the trial.

#### 2.2.1. Performance Parameters

Performance was evaluated at 21 d, which measured feed intake (FI), body weight gain (BWG) and feed conversion ratio (FCR corrected for mortality). Daily mortality and the cause of death were recorded.

#### 2.2.2. Digestibility

Excreta samples were collected by a plastic sheet being placed in each pen on top of the bedding material for 2–3 h on 3 consecutive days at the end of the trial (d 18–20). An equal amount of excreta collected on each day was pooled, mixed and used for analyses. Digestibility was calculated using titanium as the indigestible marker. Feed and excreta samples were analyzed by DM Scientific (Thirsk, UK) for DM [26], GE (Parr Bomb Calorimeter (SciMed Ltd., Cardiff, UK), using sucrose as a standard material), total nitrogen (Dumas method using a Leco Instrument (Geelen, The Netherlands)), Ca and P (EC 152/2009 Calcium AA spectrometry, Phosphorus by UV/VIS colorimetry). Estimations of digestibility were calculated for DM, AME, apparent Ca and P retention.

#### 2.2.3. Bone Analysis

Two birds per pen were euthanized by cervical dislocation on 21 d. Their tibias were removed, autoclaved at 121 °C for 15 min and were carefully defleshed of muscle and tissue by scalpel before processing. The cleaned tibias were defatted using ether extract followed by drying at 105 °C overnight and until constant weight. The bones were ashed at 650 °C for 13 h. Percentage ash was calculated as the ash weight divided by the dried bone weight. The bone ash for each tibia was then digested with aqua regia and analyzed for Ca and P content with Atomic Absorption and UV/VIS [27]. 

#### 2.2.4. Digesta Viscosity

Ileal digesta viscosity was measured on d 21, and ileal digesta were removed from two euthanized birds per pen. Digesta samples were homogenized using a plastic rod. A total of 1 g was placed in a microfuge tube and centrifuged at 5000× *g* for 10 min, and the supernatant was immediately measured for viscosity using a Brookfield Viscometer DV-I Prime (Brookfield Engineering Ltd., Harlow, UK). The measurement was taken when the reading had stabilized (10 s).

#### 2.2.5. Calculations and Statistical Analysis

Apparent total tract retentions of P and Ca were calculated using the following equation:

Nutrient retention = [1 − (Ti_diet_/Ti_excreta_) × (N_excreta_/N_diet_)] × 100

Apparent metabolizable energy (AME) (MJ/kg) was calculated using the following equation:

AME = GE_diet_ − [GE_excreta_ × (Ti_diet_/Ti_excreta_)]

Production parameter results and laboratory analyses were subjected to ANOVA (SPSS). Tukey’s HSD test was used to compare the difference between means in multiple comparison. A pen was an experimental unit. An F-ratio leading to *p* ≤ 0.05 was considered significant.

## 3. Results

The mortality during the entire study period was low, less than 2%, and was not affected by treatment (*p* > 0.05). The effect of supplementing diets with ASC on bird performance is shown in Table 3. Although the NC treatment had a numerically lower live weight at 21 days than the PC- and ASC-supplemented diets, it did not reach significance. However, the differences in these measurements, along with feed intake, were sufficient to show a significant difference in the FCR (*p* = 0.0014). The NC birds with the down-specified diet had a higher FCR than the PC birds, as expected. The addition of ASC at 200 g/t and 400 g/t improved the performance of the down-specified diet to bring it back in line with the PC FCR. Feed intake was not significantly affected during the study. 

The addition of ASC at 200 g/t and 400 g/t significantly (*p* < 0.0001) improved the viscosity of the digesta over the PC and NC. The effects of diet on the excreted DM, DM digestibility, Ca and P retention and AME are presented in Table 3. The excreted DM was significantly higher in the NC than in the ASC-supplemented diets, which is similar to the DM digestibility being significantly higher (*p* = 0.004) for the enzyme diets when compared with the NC. With the higher level of enzyme inclusion significantly improving excreted DM digestibility above the PC, P retention was significantly improved by enzyme inclusion compared to that in the PC and numerically improved compared to that in the NC. A similar pattern was seen with Ca retention; however, significance was only reached at the highest level of enzyme inclusion (*p* = 0.0007). The analyzed AME was significantly higher for all treatments compared with the NC diet (*p* < 0.0001). There were no significant differences between treatments for any of the bone measurements or mineral contents analyzed (*p* > 0.05).

## 4. Discussion 

The number of single-enzyme preparations available for the poultry industry to use to increase the digestibility and performance of their feed is relatively extensive, with a wealth of research showing that single-enzyme preparations can improve the digestibility and performance of poultry [28,29,30,31]. With numerous single enzymes routinely being added to poultry diets, it would be beneficial to reduce complexity by using a product with multi-enzyme activity which can work together within the diet. This would make diet formulation more flexible and would reduce production costs, along with decreasing digesta viscosity, enhancing nutrient digestion and improving subsequent performance. Previous studies researching the effect of dietary enzyme complexes containing residual pectinase, protease, phytase, β-glucanase, xylanase, cellulase and amylase activities have shown enhanced performance and improvements in the nutritional value of birds’ diets [32,33,34]. In a meta-analysis of 28 published broiler trials, Hooge et al. [32] reported that using a multi-enzyme product similar to ASC significantly increased body weight, improved the FCR and had an energy-sparing effect. The results of the current study, although they did not show significant improvements in body weight gain, did show a numerical improvement that resulted in a significant improvement in the FCR with the addition of the dietary enzyme complex. The lack of response in the body weight gain may be due to the relatively small, but not insignificant, down-specification of the NC and the bird only being grown to 21 days, meaning that there may not have been sufficient time to see enough of a difference in live weight. The improvement seen in this study in the FCR showed improvements in all diet treatments compared with the NC. The enzyme-supplemented diets that were down-specified for energy, Ca and P were statistically equal in FCR to the PC, indicating that the enzyme activity from the supplemented enzyme complex was able to enhance the nutritional value of the diet by increasing its digestibility.

A large percentage of broiler diets are made up of wheat, which contains anti-nutritional factor NSP. The level of NSP in wheat, and therefore poultry diets, can be detrimental to the bird’s performance [35]. NSP can increase the viscosity of the intestinal digesta, reduce nutrient digestibility and increase the FCR [36,37,38]. Therefore, it is standard practice to add enzymes, such as xylanase and β-glucanase, to a poultry diet to reduce its viscosity and improve its digestibility and performance. The dietary enzyme complex used in this study contains xylanase and residual activity of β-glucanase which are types of carbohydrases shown to reduce intestinal viscosity [39,40]. In this study, the addition of ASC at 200 g/t and 400 g/t significantly improved the viscosity of the digesta over that of the PC and NC, which may have facilitated the positive effect of the supplement on performance in this study over the NC through the enzymes’ improved nutrient digestibility. This is supported by the excreted DM being significantly higher in the NC than the enzyme-supplemented diets, which is similar to the DM digestibility being significantly higher for the enzyme diets when compared with the NC. The higher level of enzyme inclusion significantly improved excreted DM digestibility above that in the PC diet. This improvement in DM digestibility indicates that the enzyme complex was able to increase the digestibility of the diet. This may be through reducing the so-called “cage effect” [40], resulting in the release of encapsulated nutrients within cells by β-glucanase and xylanase degrading the arabinoxylans and β-glucans in the cell wall of grain. Thus, the better DM digestibility observed may be attributed to the efficiency of the enzymatic action in ASC to break down arabinoxylans and β-glucans. [41,42].

In addition to the improved viscosity and DM digestibility, this study showed a significant improvement in AME with the addition of ASC over the NC. These observed positive effects may not only be attributable to the ability of the β-glucanase and xylanase to reduce digesta viscosity and release encapsulated nutrients but may also be a result of the production of prebiotic xylo-oligosaccharides (XOSs) [43,44,45]. The wheat cell walls contain large amounts arabinoxylans (7.3% of DM whole grain) and are the main non-starch carbohydrates in wheat [46], Xylanases break down arabinoxylans in wheat-based diets, producing XOS [43]. XOS has been seen to increase SCFA-producing bacteria, the products of which can be used as a source of energy [47].

In this study, P retention from the diet was measured, and it showed that P retention was significantly improved by enzyme inclusion compared to that in the PC and increased numerically compared to that in the NC. This indicates that the residual phytase activity in the supplemented enzyme complex was able to release the bound P from the phytate in the diet to compensate for the down-specifications of P between the PC and NC diets. Similar results have been seen in other research, showing that the inclusion of phytase in broiler diets releases P bound to phytate and improves P utilization from plant-derived ingredients [48,49].

It is well known that one of the anti-nutritive effects of phytate is its ability to chelate divalent cations, such as Ca, forming stable mineral–phytate complexes and reducing their bioavailability [50,51,52]. It was shown in this study that, in addition to the improved P retention, the enzyme inclusion showed a similar pattern regarding Ca retention. This improved Ca retention with enzyme inclusion compared to that in the controls indicates that the phytase activity makes the Ca more bioavailable to the bird. This agrees with other research, which found that phytase addition improved the retention of Ca and ileal phytate P absorption, along with bone mineral density [53]. When altering Ca and P levels within broiler diets with enzyme supplementation, it is important to measure bone mineralization, as it can affect bone strength, and the ability of the bird to stand and move without hinderance [54]. In this study, we showed that reducing the Ca and P in the diet did not significantly change the analyzed mineral content of the tibia between treatments, indicating the reduced level of Ca and P within the diet was not enough to show a negative effect within the 21d trial period.

## 5. Conclusions

In conclusion, this study confirms that feeding with an enzyme complex with primary activity of phytase and xylanase can ameliorate the negative effects of the NSP and phytate present in broiler diets. This enzyme complex appears to be efficacious, supporting the performance of broilers when fed a specification with reduced amounts of Ca, P and AME, with the greatest results being seen with the higher inclusion level of ASC. Further studies are required to examine the effects of the other enzymes within ASC on broiler performance, especially the result of the combination of phytase and protease on the potential uplift in the available amino acids from the diet. The importance of enzymes in poultry feed is ever increasing to make poultry production more sustainable, both environmentally and economically. Enzymes are giving the feed industry an important tool for reducing waste and increasing the addition of co-products. Additionally, it is vital that we support birds’ gut health to improve performance and reduce the need to use antibiotics. Therefore, further studies are required to examine whether some of the positive effect seen when feeding ASC on broiler performance is due to the production of the prebiotic xylo-oligosaccharides and whether this links to a change in the microflora composition in the gut, mainly an increase in SCFA-producing bacteria.

## Figures and Tables

**Table 1:** Feed formulation of diets for broilers, 0–21 d.

Feedstuff (as % of Diet)	Positive Control ^3^	Negative Control ^4^	ASC 200 g/Mt	ASC 400 g/Mt
Wheat (CP 12.9)	37.19	40.55	41.37	41.35
Corn (CP 8.8)	20.00	20.00	20.00	20.00
Soybean meal (Hipro CP > 47.7)	33.89	33.29	32.53	32.53
Soy oil	3.83	1.88	1.82	1.82
Limestone (Fine coarse, Ca 38)	1.16	1.09	1.10	1.10
Monocalciumphosphate (Ca 17.0, P 22.7)	1.75	1.01	1.01	1.01
Salt (NaCl)	0.24	0.23	0.24	0.24
Sodium bicarbonate	0.25	0.26	0.25	0.25
Lysine HCl	0.31	0.32	0.30	0.30
DL-methionine	0.34	0.33	0.32	0.32
L-Threonine	0.16	0.16	0.16	0.16
Choline chloride (Cl 60)	0.08	0.08	0.08	0.08
Allzyme^®^ Spectrum	-	*-*	0.02	0.04
Titanium dioxide	0.40	0.40	0.40	0.40
Vitamin premix ^1^	0.20	0.20	0.20	0.20
Trace mineral premix ^2^	0.20	0.20	0.20	0.20

^1^ Supplied per kilogram: Vitamin A, 6500 IU; vitamin D3, 2500 IU; vitamin E, 40,000 mg; vitamin B12, 8.5 mg; riboflavin, 4300 mg; niacin, 30,000 mg; pantothenic acid, 8500 mg; vitamin K, 1600 mg; folic acid, 1100 mg; vitamin B6, 2700 mg; thiamine, 1600 mg; d-biotin, 150 mcg. ^2^ Supplied per kilogram: iron (ferrous sulfate), 20 mg; manganese (manganese sulfate), 120 mg; zinc (zinc oxide), 110 mg; iodine (calcium iodate), 1.25 mg; copper (copper sulphate), 16 mg; selenium (sodium selenite), 0.3 mg. ^3^ PC = feed formulation designed to mimic commercial formulation. ^4^ NC = feed formulation designed to be marginally deficient in Ca and available P, with reduced AME.

**Table 2:** Calculated nutrient contents in the experimental diet used on 0–21 d old broilers (as fed).

Nutrient (% Unless Otherwise Stated)	Positive Control	Negative Control	ASC ^1^ 200 g/Mt	ASC ^1^ 400 g/Mt
Crude Protein	23.35	23.49	23.42	23.42
Crude Fat	5.9	4	3.95	3.95
Dig Lys	1.28	1.28	1.28	1.28
Dig Met	0.64	0.64	0.63	0.63
Dig Met + Cys	0.95	0.95	0.95	0.95
Dig Trp	0.26	0.26	0.26	0.26
Dig Thr	0.86	0.86	0.86	0.86
Dig Arg	13.5	13.48	13.5	13.5
Dig Val	0.93	0.93	0.96	0.96
Dig Ile	0.86	0.86	0.87	0.87
AME, MJ/kg	12.55	12.18	12.55	12.55
Kcal/kg	3000	2911	3000	3000
Ca	0.96	0.81	0.81	0.81
Available Ca	0.96	0.81	0.96	0.96
P	0.78	0.62	0.62	0.62
Available P	0.48	0.33	0.48	0.48
Na	0.18	0.18	0.18	0.18
K	1.01	1.00	1.00	1.00
Cl	0.26	0.26	0.26	0.26
mEq/kg	300	301	296	296

^1^ ASC = Matrix values were applied giving an uplift of 129 kcal/kg, Avial. P 0.15%, Ca 0.15%.

**Table 3:** Effect of mixed-enzyme activity product on 0–21 d broiler performance, digestibility and digesta viscosity.

Parameter	Positive Control	Negative Control	ASC 200 g/Mt	ASC 400 g/Mt	SEM	*p* Value
Hatch weight (g)	42.5	42.7	42.1	42.8	0.0897	0.1552
Live weight d 21 (kg)	1.036	1.010	1.042	1.051	0.0068	0.1755
Feed intake (kg/pen)	21.29	21.04	20.57	20.39	0.1051	0.4596
FCR *	1.405 ^a^	1.449 ^b^	1.417 ^ab^	1.387 ^a^	0.0061	0.0014
Mortality (%)	1.79	1.79	0.45	1.34	0.3489	0.4928
Viscosity (cP)	6.31 ^a^	5.97 ^a^	4.04 ^b^	2.81 ^b^	0.2977	<0.0001
Excreted DM (%)	20.4 ^ab^	20.9 ^b^	19.6 ^a^	19.5 ^a^	0.1819	0.0094
Excreted DM digestibility (%)	66.6 ^ab^	66.1 ^a^	68.5 ^bc^	69.4 ^c^	0.3881	0.004
P retention (%)	50.7 ^a^	64.4 ^b^	65.8 ^b^	69.0 ^b^	1.1051	<0.0001
Ca retention (%)	53.4 ^a^	55.1 ^a^	56.7 ^ab^	60.1 ^b^	0.6349	0.0007
AME (MJ/kg)	12.64 ^a^	12.02 ^b^	12.82 ^a^	12.73 ^a^	0.0728	<0.0001
Tibia/body weight	1.50	1.48	1.48	1.49	0.0087	0.9133
Tibia ash (%)	38.0	36.8	38.4	38.5	0.2618	0.1040
Tibia ash Ca (%)	33.1	35.1	34.2	34.4	0.4657	0.5082
Tibia ash P (%)	16.3	16.8	16.4	16.7	0.1016	0.3233

* Mortality-corrected FCR; ^a–c^ means not sharing a superscript differ significantly (*p* < 0.05).

## Data Availability

The data that support the findings of this study are available from the corresponding author upon reasonable request.

## Supplementary Materials

The following supporting information can be downloaded at: https://www.mdpi.com/article/10.3390/ani14020328/s1, Table S1: Analytical composition of the experimental diets.
