# Supplementary material for: The Effect of including a Mixed-Enzyme Product in Broiler Diets on Performance, Metabolizable Energy, Phosphorus and Calcium Retention
Source: Animals (Basel). 2024 Jan 21;14(2):328. doi: 10.3390/ani14020328 (PMC10812510; doi:10.3390/ani14020328)
Supplement: Supplementary file 1 [file animals-14-00328-s001.zip › animals-2812050-supplementary.pdf]

Table S1. Analytical composition of the experimental diets.

Diet component	Positive Control	Negative Control	ASC 200 g/Mt	ASC 400 g/Mt
Dry matter (%)	87.5	87.1	87.1	87.1
Ash (g/kg dw)	77	66	72	64
Crude protein (g/kg dw)	267	275	271	274
Crude fiber (g/kg dw)	26	24	27	27
Crude fat (g/kg dw)	77	58	53	60
Titanium oxide (mg/kg dw)	4.89	4.75	4.73	4.66
